# Cardiac MRI for the prognostication of heart failure with preserved ejection fraction: A systematic review and meta-analysis

**DOI:** 10.1016/j.mri.2020.11.011

**Published:** 2021-02

**Authors:** Hosamadin Assadi, Rachel Jones, Andrew J. Swift, Abdallah Al-Mohammad, Pankaj Garg

**Affiliations:** a Department of Infection, Immunity & Cardiovascular Disease, University of Sheffield, Sheffield, UK; b Sheffield Teaching Hospital NHS Foundation Trust, Sheffield, UK

**Keywords:** Heart failure, HFpEF, Cardiac MRI, Late gadolinium enhancement, T1 mapping, Prognosis, AHA, American Heart Association, ECHO, Echocardiography, ECV, Extracellular Volume, HCM, Hypertrophic Cardiomyopathy, HF, Heart Failure, HFpEF, Heart Failure with preserved Ejection Fraction, iECV, Total Extracellular Volume of the myocardium indexed to body surface area, LGE, Late Gadolinium Enhancement, LVEDV, Left Ventricular End-Diastolic Volume, LVEF, Left Ventricular Ejection Fraction, MACE, Major Adverse Cardiovascular Events, MI, Myocardial Infarction, MOLLI, Modified Look-Locker Inversion recovery, MRI, Magnetic Resonance Imaging, NICM, Non-Ischaemic Cardiomyopathy, NYHA, New York Heart Association, PRISMA, Preferred Reporting Items for Systematic Reviews and Meta-Analyses, PROSPERO, International Prospective Register of Systematic Reviews, ROC, Receiver Operating Characteristic curve, RVEF, Right Ventricular Ejection Fraction, RVSD, Right Ventricular Systolic Dysfunction, TTE, Transthoracic Echocardiography, VT, Ventricular Tachycardia

## Abstract

**Background:**

Cardiac magnetic resonance imaging (MRI) is emerging as an important imaging tool in the assessment of heart failure with preserved ejection fraction (HFpEF). This systematic review and meta-analysis aim to synthesise and consolidate the current literature on cardiac MRI for prognostication of HFpEF.

**Methods design:**

Systematic review and meta-analysis. Data sources: Scopus (PubMed and Embase) for studies published between 2008 and 2019. Eligibility criteria for study selection were studies that evaluated the prognostic role of cardiac MRI in HFpEF. Random effects meta-analyses of the reported hazard ratios (HR) for clinical outcomes was performed.

**Results:**

Initial screening identified 97 studies. From these, only nine (9%) studies met all the criteria. The main cardiac MRI methods that demonstrated association to prognosis in HFpEF included late gadolinium enhancement (LGE) assessment of scar (*n* = 3), tissue characterisation with T1-mapping (*n* = 4), myocardial ischaemia (*n* = 1) and right ventricular dysfunction (RVSD) (n = 1). The pooled HR for all 9 studies was 1.52 (95% CI 1.05–1.99, *P* < 0.01). Sub-evaluation by cardiac MRI methods revealed varying HRs: LGE (net *n* = 402, HR = 1.6, 95% CI 0.42–2.78, *P* = 0.008); T1-mapping (*n* = 1623, HR = 1.25, 95% CI 0.891–1.60, *P* < 0.001); myocardial ischaemia or RVSD (*n* = 325, HR = 3.19, 95% CI 0.30–6.08, *P* = 0.03).

**Conclusion:**

This meta-analysis demonstrates that multiparametric cardiac MRI has value in prognostication of patients with HFpEF. HFpEF patients with a detectable scar on LGE, fibrosis on T1-mapping, myocardial ischaemia or RVSD appear to have a worse prognosis.

**PROSPERO registration number:**

CRD42020187228.

## 1 Introduction

Heart failure with preserved ejection fraction (HFpEF) accounts for over 50% of all heart failure cases [1]. HFpEF is a clinical syndrome in which the heart is incapable of delivering enough oxygen to the tissues proportionate with their metabolic demands while the contraction is not impaired [2]. It is associated with poor quality of life, significantly more need for medical attention, and a higher risk of premature death [3]. HFpEF's pathophysiology is still broadly unknown, which partly explains why there is no specific treatment for it. The prevalence of HFpEF is increasing and leading to an emerging epidemic with increased morbidity and mortality ranging from 10% to 30% annually and is higher in epidemiological studies than in clinical trials. The major causes of death in HFpEF are cardiovascular, making up 51–60% of deaths in epidemiological studies and > 70% in clinical trials [4].

**Fig. 1:**
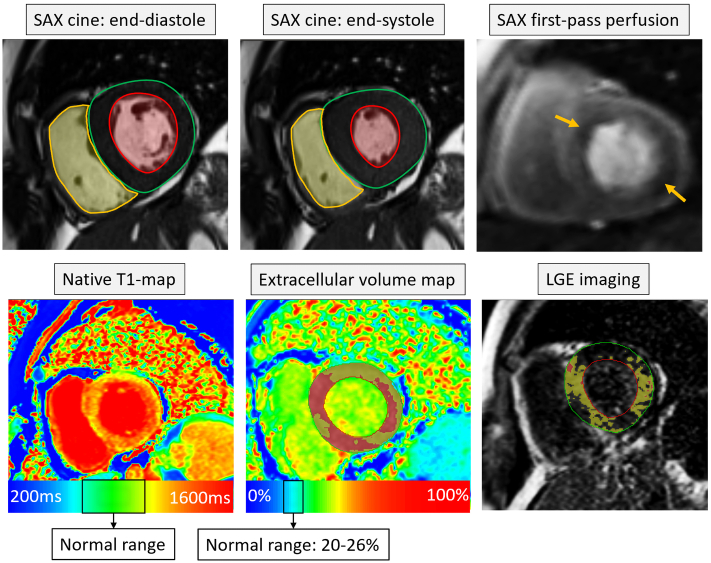
Multiparametric cardiac magnetic resonance imaging in patients with HFpEF. In this particular case, there is evidence of concentric left ventricular hypertrophy with an LV ejection fraction of 62%. Also, there is evidence of perfusion defect during first-pass perfusion (orange arrows). T1-mapping shows a global pattern of rise in values. ECV mapping demonstrates large areas of the myocardium with an ECV of 30% to 50% (yellow area) and also >50% (red areas of the myocardium). A similar pattern of fibrosis was seen on LGE imaging. (For interpretation of the references to colour in this figure legend, the reader is referred to the web version of this article.)

Although transthoracic echocardiography (TTE) remains the primary diagnostic tool for HFpEF, cardiac magnetic resonance imaging (MRI) is the recognised gold standard for the assessment of left ventricular (LV) systolic function, confirming left atrial enlargement and LV hypertrophy [5]. In addition to volumetric assessment, multiparametric cardiac MRI tissue characterisation and first-pass perfusion can further sub-phenotype patients with HFpEF [6]. This is becoming more relevant as for certain HFpEF sub-phenotypes, there are emerging therapies [7]. Late gadolinium enhancement (LGE) imaging by cardiac MRI is a valuable tool for scar detection [8]. Beyond focal scar lesions, illustrated by LGE imaging methods, native and post-contrast T1 mapping can recognise diffuse myocardial fibrosis, previously not feasible by noninvasive techniques (Fig. 1) [9]. Myocardial ischaemia assessment by first-pass perfusion cardiac MRI is non-inferior to invasive quantitative ischaemia evaluation with respect to major adverse cardiac events (MACE) [10].

Previously published studies have focused more on the diagnostic value of cardiac MRI in HFpEF, while the overall prognostic utility of cardiac MRI remains unclear. In addition, it is not clear which cardiac MRI method has the most evidence to guide prognosis in HFpEF. Systematic evidence synthesis is required to develop further understanding of which cardiac MRI methods are clinically applicable to predict prognosis in HFpEF. The aim of this systematic review and meta-analysis was, therefore, to summarise current literature, systematically consolidate studies which have evaluated the role of cardiac MRI in prognostication of HFpEF.

## 2 Methods

### 2.1 Systematic review and meta-analysis registration

This project was prospectively registered (CRD42020187228) [11] with the prospective register of systematic review (PROSPERO), the international database of prospectively registered systematic reviews and meta-analyses in health, where there is a health-related outcome.

### 2.2 Eligibility criteria

Studies which assessed the prognostic significance of cardiac MRI in HFpEF were included. The primary outcomes of interest were HF hospitalisation and all-cause mortality. This systematic review and meta-analyses mainly focused on the hazard ratio (HR) for outcomes, and all subsequent meta-analyses were planned using HR and standard error values. We limited our search to full articles, medicine, and human participants. Studies that were not published in English were excluded. Ethics approval was not required, as no patients were involved.

### 2.3 Search strategy and study selection

The literature search was done on the Scopus database. This database incorporates all Medline and Embase results. The initial screening was done by HA, RJ and PG on 21/11/2019, followed by a second screening on 15/10/2020, and included the following terms: (TITLE-ABS-KEY (heart AND failure AND with AND preserved AND ejection AND fraction) OR TITLE-ABS-KEY (hfpef) AND TITLE-ABS-KEY (cardiac AND magnetic AND resonance AND imaging) OR TITLE-ABS-KEY (cardiac AND mri) AND TITLE-ABS-KEY (prognosis) OR TITLE-ABS-KEY (outcome)). We also searched manually the reference lists of relevant studies. All searches were integrated and duplicates were removed.

**Fig. 2:**
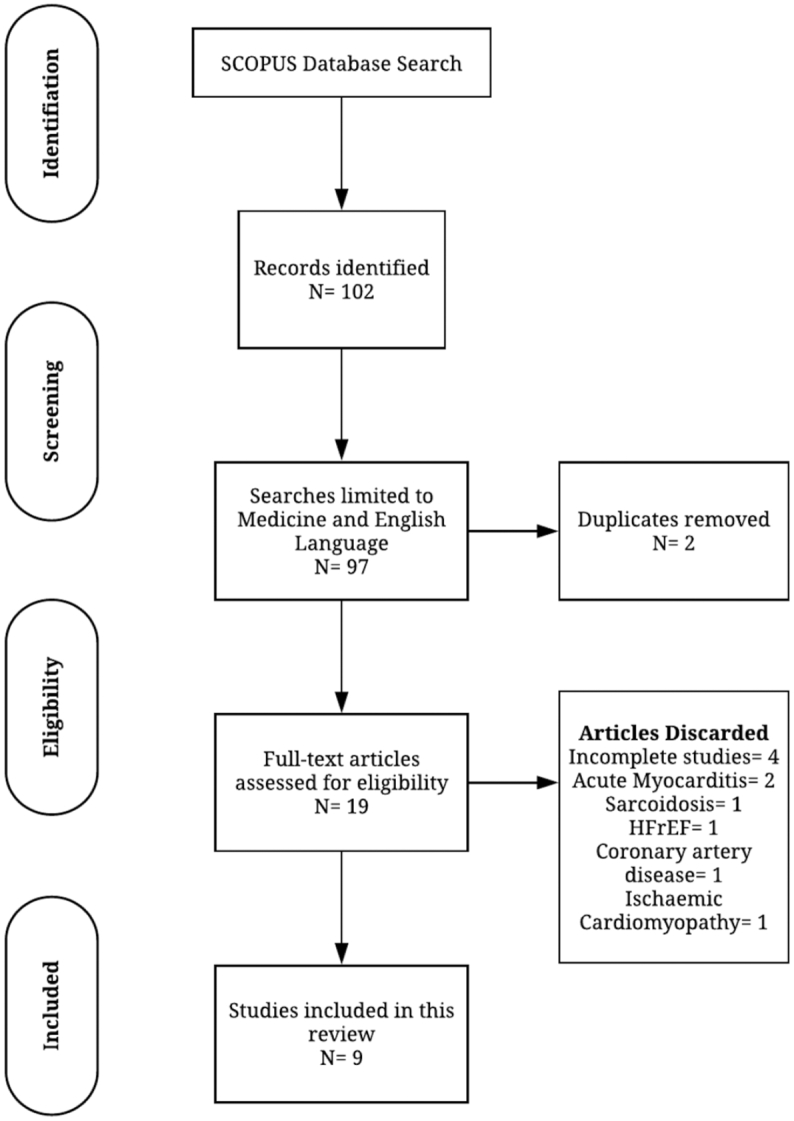
Flow diagram demonstrating evidence synthesis for the systematic review, adapted from Moher et al. 2009 [12] using the PRISMA tool.

PRISMA guidelines were used for the identification, screening, inclusion and selection of studies. The final shortlist of included studies was reviewed and approved by two independent assessors, PG and AAM. Fig. 2 details the search strategy, using the Preferred Reporting Items for Systematic Reviews and Meta-Analyses (PRISMA) tool [12].

### 2.4 Statistical analysis

Statistical analysis was performed using MedCalc (MedCalc Software, Ostend, Belgium version 19.1.5). Graphing was undertaken in Origin Lab Pro (Origin Lab Corp., Northampton, MA), and Lucidchart diagram (Lucid Software Inc., Utah, USA). We pooled the values of HR for clinical outcomes and the number of patients recruited in each study. The inverse variance method was used to calculate the weighted summary HRs under the random-effects model. In the inverse variance method, the weight given to each study is the inverse of the variance of the effect estimate (i.e. one over the square of its standard error). Thus, larger studies are given more weight than smaller studies, which have larger standard errors. This choice of weight minimises the imprecision (uncertainty) of the pooled effect estimate. Next, the heterogeneity statistics were incorporated to calculate the summary correlation coefficient under the random-effects model with 95% confidence interval (CI) was used in all analyses. The main findings of the meta-analysis were presented as Forest plots and tables. We used the I^2^ statistic to measure heterogeneity among the trials in each analysis and used a threshold of 75% as significantly heterogeneous.

## 3 Results

After an initial screening of 97 research studies, nine studies were identified and included in the systematic review and meta-analysis. For each study, we identified the number of patients involved; the primary cardiac MRI method used; the follow-up period and the clinical outcomes with the hazard ratio for cardiac MRI variables to predict outcomes. From the initial results, 88 studies were excluded for not meeting our inclusion criteria. Seven included studies were prospective single-centre observational cohorts [6,[13], [14], [15], [16], [17], [18]], and two studies were retrospective single-centre observational cohorts [19,20]. Analysts in all studies were blinded to cardiac MRI results. The mean age was 63 ± 21, and 1084 (46%) patients were males. The total number of patients included was 2350, and the median follow up period ranged between 7.5 and 48 months. The assessed clinical outcomes included heart failure hospitalisations, cardiac transplantations, and death. A summary of baseline characteristics, outcomes, cardiac MRI metrics, and their significance of the nine studies included in the systematic review and meta-analyses is detailed in Table 1 and Table 2.

**Table 1:** Summary of baseline characteristics and outcomes of studies included in the systematic review and meta-analysis.

First author	Year	N	Age (yrs)	Male sex	LVEF %	FU	Clinical outcome
Late gadolinium enhancement scar imaging							
Murtagh G.	2016	205	56 ± 7	64 (31)	61 ± 6	7.5	8 deaths and 4 HF hospitalisations
Kato S.	2015	111	70 ± 14	56 (50)	61 ± 10	28.3 ± 20.3	2 deaths and 6 HF hospitalisations
Pöyhönen P.	2014	86	52 ± 10	47 (55)	50	27.8	13 deaths and 2 cardiac transplantations

T1-mapping and other relaxometry techniques							
Kanagala P.	2019	232	73 ± 8	67 (49)	56 ± 6	47.6	14 deaths and 28 HF hospitalisations
Schelbert E.B.	2017	1174	55 ± 11	637 (54)	62	23.1	48 deaths and 13 HF hospitalisations
Duca F.	2016	117	74 ± 8	36 (31)	64 ± 10	24	4 deaths and 30 HF hospitalisations
Mascherbauer J.	2013	100	70 ± 7	38 (40)	64 ± 11	22.9 ± 5	3 deaths and 13 HF hospitalisations

Others CMR methods including ischaemia assessment							
Kanagala P.	2018	154	72 ± 10	78 (51)	57 ± 6	20.7	19 deaths and 43 HF hospitalisations
Aschauer S.	2016	171	70 ± 9	61 (35)	63 ± 9	19.1 ± 12.9	15 deaths and 26 HF hospitalisations

Abbreviations: FU, follow-up (in months); HF, heart failure; LVEF, left ventricular ejection fraction.

**Table 2:** Summary of Cardiac MRI metric and their significance for studies included in this systematic review and meta-analysis.

First author	Cardiac MRI metric	HR, 95% CI	*P*-value
Late gadolinium enhancement scar imaging			
Murtagh G.	LGE	1.14, 1.09–1.18	<0.01
Kato S.	LGE	7.913, 1.603–39.05	0.012
Pöyhönen P.	LGE	1.027, 1–1.04	0.003

T1-mapping and other relaxometry techniques			
Kanagala P.	iECV	1.689, 1.141–2.501	0.009
Schelbert E.B.	ECV	1.75, 1.25–2.45	0.001
Duca F.	ECV	1.099, 1.005–1.201	0.038
Mascherbauer J.	Native-T1	0.99, 0.98–0.99	0.046

Others CMR methods including ischaemia assessment			
Kanagala P.	Stress cardiac MRI	1.92, 1.07–3.45	0.03
Aschauer S.	RVSD	4.90, 2.46–9.75	<0.001

Abbreviations: CI, confidence interval; ECV, extracellular volume; HR, hazard ratio; iECV, total extracellular volume of the myocardium indexed to body surface area; LGE, late gadolinium enhancement; MOLLI, modified look-locker inversion; MRI, magnetic resonance imaging; RVSD, right ventricular systolic dysfunction.

### 3.1 Meta-analysis

**Fig. 3:**
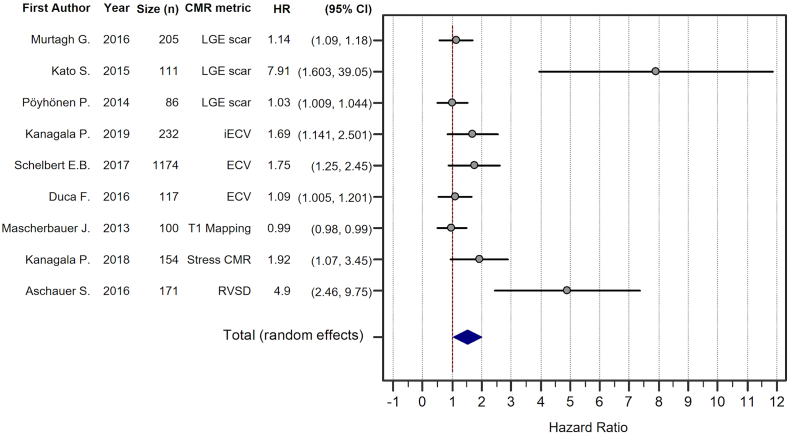
Forest plot of pooled hazard ratio (HR) for all the studies with weighted random-effects model HR (1.518, 95% CI 1.05 to 1.99) in favour of cardiac MRI assessment for prognostication of HFpEF patients.

The studies were divided into three groups by cardiac MRI method: late gadolinium enhancement scar imaging, T1 mapping and other relaxometry techniques, and other cardiac MRI methods including ischaemia assessment. The pooled analysis across all cardiac MRI methods showed a total random effect of 1.52 (SE = 0.24, 95% CI 1.05–1.99, *P* < 0.001, z = 6.3) (Fig. 3). The strongest study was that by Kato et al. [13], who recruited 111 patients and found a high HR of 7.91 (SE = 2.01, 95% CI 3.96–11.87). This study was included in the LGE group, which overall had a total random effect of 1.60 (SE = 0.60, 95% CI 0.42–2.78, *P* = 0.008, z = 2.66).

The second group consisted of four studies focusing on tissue characterisation and had a total random effect of 1.25 (SE = 0.18, 95% CI 0.891–1.60, *P* < 0.001, z = 6.91). The study with the lowest hazard ratio was that by Mascherbaur et al. [14] (HR = 0.99, SE = 0.25, 95% CI 0.50–1.49). The study with the highest HR was that by Schelbert et al. [15] (HR = 1.75, SE = 0.45, 95%CI 0.88–1.63) and was also the largest study of the whole cohort, recruiting 1174 patients.

The final group consisted of two studies using cardiac MRI to assess either myocardial ischaemia or right ventricular function. The total random effect was the highest of the three groups, at 3.19 (SE = 1.47, 95% CI 0.30–6.08, *P* = 0.03, z = 2.16).

A strong correlation between the right heart and prognosis in HFpEF was reported by Aschauer et al. [16]. Of the 171 patients with HFpEF, 33 patients (19.3%) had right ventricular systolic dysfunction (RVSD) (right ventricular ejection fraction [RVEF] <45%) by cardiac MRI and were followed up for (19.1 ± 12.9) months. During this time, 19 of these patients (57.6%) reached the primary endpoint (death/HF hospitalisation). In comparison with patients without RVSD, by univariable and multivariable Cox analysis, RVSD was markedly linked with reduced event-free survival (HR = 4.90, 95% CI 2.46–9.75, *P* < 0.001). Nested Cox regression models established the mortality rate of RVSD by cardiac MRI (HR 5.05, 95% CI 1.82–13.95, *P* = 0.002). RVSD was the most potent prognosticator of death.

## 4 Discussion

This meta-analysis demonstrated that cardiac MRI derived markers can predict the prognosis and outcome of patients with HFpEF by unmasking potential clinically undiagnosed underlying heart diseases, and identifying structural changes in the myocardium that could adversely affect the prognosis in several patients. These markers include myocardial late gadolinium enhancement usually signifying myocardial scarring, tissue characterisation by native and post-contrast T1 mapping which usually detect myocardial fibrosis, stress cardiac MRI which detects underlying myocardial ischaemia, and volumetric right ventricular assessment on cines for RVSD.

### 4.1 Late gadolinium enhancement scar imaging

LGE for prognostication in HFpEF was first introduced in 2015 by Kato et al. [13]. They found LGE in 40 patients (36%) of their 111 patients. During follow up, 10 of the 111 patients (9%) experienced major adverse cardiovascular events, including death. Interestingly, eight of these ten patients were in the LGE+ group. Assessing the predictors of mortality by multivariate Cox proportional hazard analysis, they found that percentage of scar by LGE imaging independently predicted future events after the adjustment with age, presence of DM, New York Heart Association (NYHA) classification, history of HF hospitalisation, and LVEF which could explain the high HR. They demonstrated that 6% burden of LV scar tissue had a sensitivity of 80% and specificity of 77% to predict events. The Kaplan-Meier curves stratified by the presence and size of LGE demonstrated a significant impact on the prognosis of patients with HFpEF (*P* = 0.016 by Log-rank test).

Murtagh et al. [20] used cardiac MRI derived LGE in the cardiac risk stratification of patients with extracardiac sarcoidosis. LGE was present in 41 patients (20%). Among the 205 patients in the cohort, 12 patients (6%) died or had ventricular tachycardia (VT), 10 of these 12 patients (83%) were in the LGE+ group. Murtagh et al. estimated that the death/VT rate per year was 20% higher in the LGE+ group than the LGE− group (4.9% vs 0.2%, *P* < 0.01). The percentage of scar quantified by LGE that predicted the risk of death/VT events and for recognising patients with myocardial damage despite having a preserved ejection fraction was 5.7%, with sensitivity and specificity of 87% and 62% respectively. Interestingly, their findings are similar to the findings by Kato et al.

In another study, Pöyhönen et al. [19] evaluated the value of LGE imaging in patients suspected with non-ischaemic cardiomyopathy (NICM). Even though this study was not directly in patients with HFpEF, it was included in this systematic review as the mean left ventricular ejection fraction (LVEF) was 52%. In this study, the event rate for MACE was 26% in patients with LGE+ versus 4% in patients without LGE (*P* = 0.041). Of the 86 patients involved, 15 reached the endpoint (17%), with an event rate of 7.6% per year. The highest event rate was observed in patients with LGE volume of ≥17%. Thus, the presence of LGE while not essential in the cardiac MRI diagnosis of HFpEF appears to define the patients with a higher risk of major cardiovascular events, including death (Table 3).

**Table 3:** Results of group wise meta-analysis of cardiac MRI methods.

Study	HR	SE	95% CI	z	P	Weight (%)
Late gadolinium enhancement scar imaging						
Murtagh G.	1.14	0.291	0.570 to 1.710			45.75
Kato S.	7.913	2.019	3.956 to 11.870			7.55
Pöyhönen P.	1.027	0.262	0.514 to 1.540			46.7
Total (fixed effects)	1.141	0.194	0.761 to 1.520	5.887	<0.001	100
Total (random effects)	1.598	0.601	0.421 to 2.776	2.662	0.008	100
Test for heterogeneity						
Q	11.4438					
DF	2					
Significance level	*P* = 0.0033					
I2 (inconsistency)	82.52%					
95% CI for I2	46.43 to 94.30					

T1-mapping and other relaxometry techniques						
Kanagala P.	1.689	0.431	0.845 to 2.533			15.61
Schelbert E.B.	1.75	0.446	0.875 to 2.625			14.65
Duca F.	1.099	0.28	0.549 to 1.649			32.12
Mascherbauer J.	0.99	0.253	0.495 to 1.485			37.62
Total (fixed effects)	1.221	0.161	0.906 to 1.536	7.606	<0.001	100
Total (random effects)	1.245	0.18	0.892 to 1.599	6.906	<0.001	100
Test for heterogeneity						
Q	3.6099					
DF	3					
Significance level	*P* = 0.3068					
I2 (inconsistency)	16.90%					
95% CI for I2	0.00 to 89.27					

Others CMR methods including ischaemia assessment						
Kanagala P.	1.92	0.49	0.960 to 2.880			57.45
Aschauer S.	4.9	1.25	2.450 to 7.350			42.55
Total (fixed effects)	2.317	0.456	1.423 to 3.210	5.08	<0.001	100
Total (random effects)	3.188	1.473	0.300 to 6.076	2.164	0.03	100
Test for heterogeneity						
Q	4.927					
DF	1					
Significance level	*P* = 0.0264					
I2 (inconsistency)	79.70%					
95% CI for I2	12.50 to 95.29					

Abbreviations: CI, confidence interval; DF, degree of freedom; FU, follow-up (in months); HR, hazard ratio; MRI, magnetic resonance imaging; Q, chi-squared statistic; SE, standard error.

### 4.2 T1 mapping and other relaxometry techniques

While LGE can only identify focal fibrosis, cardiac MRI T1 mapping can uncover and quantify diffuse fibrosis and has the potential to identify focal fibrosis as well in the myocardium [[21], [22], [23]]. Both native and post-contrast T1 mapping has proved reliability in the diagnosis of cardiomyopathies; in predicting their prognosis, and in directing their further treatment. Schelbert et al. [15] that increased extracellular volume (ECV) was associated with a high mortality rate in patients with HFpEF (HR = 1.75 per 5% increase in ECV, 95% CI 1.25–2.45, *P* = 0.001). Mascherbauer et al. [14] abnormal cardiac MRI T1 mapping in 61 patients with HFpEF. During follow up, 16 (26.2%) patients reached the primary endpoint (three patients died, and 13 had HF hospitalisations). Post-contrast T1 time was associated with the endpoint (HR = 0.99, 95% CI 0.98–0.99, *P* = 0.046). In histopathology, T1 time notably corresponded with extracellular matrix area in LV biopsy samples taken from patients and was strongly linked with the outcome.

Duca et al. [17], measured myocardial ECV by cardiac MRI T1 mapping using the modified look-locker inversion recovery (MOLLI) sequence and validated it against histology in a subset of their patient's cohort for the prognostication of HFpEF. During follow-up for a median of 24 months, 34 (29%) of 117 patients reached the primary endpoint (four deaths and 30 HF hospitalisations). By multivariable Cox regression analysis, MOLLI-ECV was associated with outcome among imaging variables (HR = 1.099, 95% CI 1.01–1.2, *P* = 0.038). Moreover, the histological ECV (30.1 ± 4.6%) significantly correlated with MOLLI-ECV (*r* = 0.494, *P* = 0.037). In their study, patients with higher MOLLI-ECV had shorter event-free survival (Log-rank test: *P* = 0.028). Kanagala et al. [18] studied 140 patients (96 HFpEF, 44 controls) using cardiac MRI. 42 (44%) of 96 HFpEF patients reached the primary endpoint (14 deaths, 28 hospitalisations). They used indexed ECV, which is the product of the percentage of ECV multiplied by the indexed left ventricular end-diastolic volume (LVEDV) (iECV = ECV (%) × indexed LVEDV). HFpEF patients had greater diffuse fibrosis (iECV = 13.7 ± 4.4 ml/m^2^) than controls (iECV = 10.9 ± 2.8 ml/m^2^) (*P* < 0.0001). In multivariate analysis, iECV was an independent predictor of outcome (HR = 1.689, 95% CI 1.141–2.501, *P* = 0.009). Overall, 92 (33.5%) of 274 recruited patients for cardiac MRI prognostication reached the primary endpoint.

Therefore, the detection of diffuse myocardial fibrosis by increased T1 or increased ECV are markers of major cardiovascular events and death in patients with HFpEF (Table 3). Nevertheless, more evidence and standardisation is warranted for a clearer clinical translation of quantitative mapping techniques for further characterisation of the myocardium.

### 4.3 Stress cardiac MRI

Kanagala et al. [6] observed cardiac MRI's superiority over echocardiography (TTE) in detecting hidden pathology among 154 patients with HFpEF using first-pass stress perfusion cardiac MRI. Having excluded patients with known cardiac and non-cardiac pathology that could adversely affect the prognosis, 42 (27%) patients had an undiagnosed pathology. These included - coronary artery disease [20], microvascular dysfunction [11], hypertrophic cardiomyopathy [10], and constrictive pericarditis [5]. Patients with HFpEF who had newly added diagnoses had a worse prognosis during follow up (Log-rank test, *P* = 0.047). 53 of 154 patients reached the primary endpoint, in which 20 (38%) of the 53 patients were of the ‘newly diagnosed pathology’ group by cardiac MRI. On multivariate Cox proportional hazards analysis, patients with a new diagnosis had the most powerful predictors of outcome (HR = 1.92, 95% CI = 1.07–3.45, *P* = 0.03).

In addition to myocardial ischaemia, the use of stress cardiac MRI uncovered associated pathology in patients with HFpEF, which identified patients with a higher risk of major cardiovascular events, including death (Table 3).

### 4.4 Right ventricular systolic dysfunction (RVSD)

A strong correlation between right ventricular systolic dysfunction (RVEF <45%) and prognosis in HFpEF was reported by Aschauer et al. [16]. Using univariable and multivariable Cox analysis, RVSD was markedly linked with reduced event-free survival (HR = 4.90, 95% CI 2.46–9.75, *P* < 0.001).

### 4.5 Clinical perspective

This systematic review and meta-analysis identified LGE, T1 mapping, stress cardiac MRI and detection of RVSD as four cardiac MRI derived markers of risk of MACE and mortality in patients with HFpEF. These, along with the robust characteristics of the diagnostic abilities of cardiac MRI do call for re-configuration of the workup of patients with HFpEF.

Cardiac MRI provides the capabilities not only of confirming that the left ventricular ejection is preserved along with inevitable dilatation of the left atrium seen in patients with HFpEF; it provides information about possible aetiology or other by-stander pathologies that could alter the prognosis. It detects scarring known to be associated with increased risk of arrhythmia and may predict reduced responsiveness to therapeutics of heart failure. The systemic review and meta-analysis identified a threshold for LGE beyond which there is an increased risk of MACE and mortality (Table 4). Similarly, the detection of diffuse fibrosis by T1 mapping was demonstrated to be associated with increased risk of MACE, including mortality, particularly once a certain threshold was crossed (Table 4). Other findings such as ischaemia and RVSD were proved to be vital additions in the prediction of poor outcome.

**Table 4:** Summary of clinically relevant cut-offs for several cardiac MRI metrics which have prognostic significance.

First Author	Cardiac MRI metric	Thresholds for prognosis	Quantification and segmentation methods
Murtagh G. 2016	LGE	LGE volume 5.7%	Signal intensity >5 SD above the mean signal intensity of remote myocardium
Kato S. 2015	LGE	LGE volume 6%	Signal intensity >2 SD above the mean signal intensity of a remote myocardium
Pöyhönen P. 2014	LGE	LGE volume ≥ 17%	Visual scoring on AHA 17-segment model of the LV
Kanagala P. 2019	iECV\ECV	iECV >16.8 ml/m2 ECV >30.7	iECV = ECV (%) × indexed LVEDV
Schelbert E.B. 2017	ECV	ECV >29%	ECV = λ × (1 − haematocrit), where λ = (ΔR1myocardium)/(ΔR1bloodpool)
Duca F. 2016	ECV	ECV >28.9%	ECV = λ × (1 − haematocrit), where λ = (ΔR1myocardium)/(ΔR1bloodpool)
Mascherbauer J. 2013	Post-contrast T1	T1 time < 388.3 ms	Measured in undefined myocardium
Kanagala P. 2018	Tissue characterisation + Stress CMR	Perfusion defect [24]	Full clinical assessment leading to new diagnosis
Aschauer S. 2016	RVSD	RV EF <45%	Standard volumetric contours

Abbreviations: AHA, american heart association; CI, confidence interval; ECV, extracellular volume; FU, follow-up (in months); HR, hazard ratio; iECV, total extracellular volume of the myocardium indexed to body surface area; LGE, late gadolinium enhancement; LV, left ventricle; LVEDV, left ventricular end-diastolic volume; MRI, magnetic resonance imaging; RVEF, right ventricle ejection fraction; RVSD, right ventricular systolic dysfunction; SD, standard deviation.

The heterogeneity of LGE imaging makes it less attractive for the clinical translation in prognostication of patients with HFpEF. However, in our study, tissue mapping techniques, which inherently are quantitative methods, demonstrate lower heterogeneity and are more appropriate for clinical translation. This does not come as a surprise bearing in mind they remove the heterogeneity of the qualitative imaging data.

Thus, we believe that cardiac MRI should be routinely considered as an essential tool in the workup of patients diagnosed with HFpEF to at least inform the prognosis and potentially to select patients in the future to be enrolled in studies of therapeutics designed to reduce the risk of heart failure hospitalisation.

### 4.6 Limitations

In this systematic review and meta-analysis, we did not include studies specifically looking at the diagnostic accuracy of any cardiac MRI metric for HFpEF. Hence, we did not report studies which have done sensitivity/specificity analysis. Caution should be applied in the judgement of meta-analysis where heterogeneity was greater than 75%. Future studies need a clearer framework for acquisition and post-processing to limit this heterogeneity for clinical translation. Due to the lack of significant number of studies representing stress cardiac MRI and right ventricular assessment for prognostication in HFpEF, we have combined them into a ‘heterogenous’ group. The results of this heterogeneous group are only hypothesis generating and caution should be applied for considerable judgement of the results. More studies are warranted exploring multi-parametric cardiac MRI utility in prognostication of patients with HFpEF.

## 5 Conclusion

This meta-analysis demonstrates that multiparametric cardiac MRI has value in prognostication of patients with HFpEF. HFpEF patients with a detectable scar on LGE, fibrosis on T1-mapping, myocardial ischaemia or RVSD appear to have a worse prognosis.

### 5.1 Key messages

What is already known about this subject?

● Cardiac magnetic resonance imaging (MRI) is emerging as an important imaging tool in the assessment of heart failure with preserved ejection fraction (HFpEF).

What does this study add?

● Evidence gathered in this systematic review and meta-analysis demonstrates that multiparametric cardiac MRI has value in the prognostication of patients with HFpEF.
● HFpEF patients with a detectable scar on LGE, fibrosis on T1-mapping, myocardial ischaemia or RVSD appear to have a worse prognosis.

How might this impact on clinical practice?

● Cardiac MRI should be considered in the diagnostic work-up of patients with suspected HFpEF. Cardiac MRI not only allows for better sub-phenotyping of HFpEF, but also to risk stratify HFpEF patients depending on cardiac MRI characteristics.

## Ethics approval and consent to participate

Ethics approval was not required, as no patients were involved.

## Consent for publication

All authors have read and approved the final version of this manuscript.

## Availability of data and materials

The datasets used and/or analysed during the current study are available from the corresponding author on reasonable request.

## Author contributions

AAM, AJS and PG conceived the idea and need for the systematic review. HA, RJ and PG did the literature search. HA and PG drafted the initial version. AJS, AAM and PG provided expert critical input to the content independently.

## Funding

This work was supported by the 10.13039/100010269Wellcome Trust (AJ: 205188/Z/16/Z). PG is supported by Academy of Sciences Starter Grant (PG: SGL018\1100) and by Wellcome Trust (PG: 220703/Z/20/Z).

## Declaration of Competing Interest

The authors declare that they have no competing interests.
